# Multimodality Imaging Evaluation of Primary Right Atrial Paraganglioma: A Case Report and Literature Review

**DOI:** 10.3389/fmed.2022.942558

**Published:** 2022-06-30

**Authors:** Wen-peng Huang, Ge Gao, Zhao Chen, Yong-kang Qiu, Jian-bo Gao, Lei Kang

**Affiliations:** ^1^Department of Nuclear Medicine, Peking University First Hospital, Beijing, China; ^2^Department of Radiology, Peking University First Hospital, Beijing, China; ^3^Department of Radiology, The First Affiliated Hospital of Zhengzhou University, Zhengzhou, China

**Keywords:** heart neoplasms, 18F-FDG, PET/CT, magnetic resonance imaging, x-ray computed tomography, paraganglioma, case report

## Abstract

**Background:**

Cardiac paraganglioma (CPGL) accounts for 1–3% of cardiac tumors and is usually benign. In total, 35–50% of CPGL lesions secrete catecholamines, causing hypertension, excessive sweating, palpitations, headache, and other symptoms. Preoperative imaging evaluation is important to determine the location of the cardiac mass, its blood supply vessels, and the relationship with surrounding structures. Multimodal imaging techniques combine with morphological and functional information to provide powerful methods for preoperative diagnosis and lesion localization. Furthermore, they can assist to reduce the incidence of intraoperative and postoperative complications and improve patient prognosis.

**Case Report:**

A 67-year-old woman suffered from paroxysmal palpitations with a heart rate of 110 beats per minute 1 month ago. Urine catecholamine and methoxyepinephrine levels were significantly increased. The patient had a 5-year history of hypertension with a maximum blood pressure of 160/100 mmHg. Computed tomography (CT) examination found a soft tissue mass in the right atrium with heterogeneous and significant enhancement, whose blood supply was from the left ileal branch artery. The patient then underwent cardiac magnetic resonance (CMR). The lesion showed inhomogeneous iso signals on the T1-weighted image (T1WI), slightly high signals on the T2 fat-suppression image, inhomogeneous high signals on the diffusion-weighted imaging (DWI), and apparent diffusion coefficient (ADC) images. The mass exhibited heterogeneous and significant enhancement on the first perfusion and delayed scans after intravenous contrast injection. However, abnormal signals were surprisingly found in the patient’s right lung, and the possibility of metastatic lesions could not be excluded. The patient underwent F-18 fluorodeoxyglucose-positron emission tomography/computed tomography (^18^F-FDG PET/CT) to rule out metastatic lesions. A fluorodeoxyglucose (FDG)-avid soft tissue mass was shown in the right atrium, with the maximum standardized uptake value (SUVmax) at about 15.2, as well as a pathological intake of brown fat throughout the body. Combined with clinical symptoms, CPGL was considered without significant sign of metastasis in ^18^F-FDG PET/CT. Finally, the patient underwent surgical resection and the post-operative pathology confirmed a CPGL.

**Conclusion:**

The combination of ^18^F-FDG PET/CT with the CMR containing different image acquisition sequences provides a powerful aid for preoperative non-invasive diagnosis, localization, and staging of CPGL, which helps to reduce intraoperative and postoperative complications and improve patient prognosis.

## Introduction

Primary cardiac tumors are very rare, with a prevalence ranging from 0.001 to 0.03%, of which about 20–30% are malignant (1, 2). Cardiac paraganglioma (CPGL) is extremely rare as it only accounts for 1–3% of cardiac tumors. In total, 35–50% of CPGL lesions secrete catecholamines, causing hypertension, excessive sweating, palpitations, headache, and other symptoms, most of which can be cured by complete surgical resection. Preoperative imaging evaluation is important to determine the exact location of CPGL, the blood supply vessels, and the relationship with surrounding structures. The use of multimodality imaging techniques, combined with morphological and functional information, is helpful in the preoperative diagnosis, localization of tumors, reduction of the incidence of intraoperative and postoperative complications, and improvement of patient prognosis.

## Case Presentation

**FIGURE 1:**
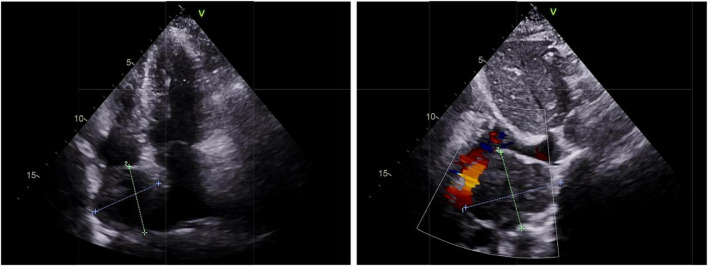
Echocardiography of cardiac paraganglioma. An enlarged left atrium and a hypoechoic mass of about 4.6 cm × 4.6 cm at the top of the right atrium near the superior vena cava was found with clear borders and uniform internal echogenicity, as well as the compression of the right atrium and the superior vena cava.

A 67-year-old woman came to the hospital with 1-month paroxysmal palpitations and 1-week bilateral lower limb edema with aggravation for 2 h. The patient presented with paroxysmal palpitations with a heart rate of 110 beats per minute 1 month ago and received no treatment. The patient had a 5-year history of hypertension and a 4-year history of diabetes mellitus, with a maximum blood pressure of 160/100 mmHg, and usually took oral antihypertensive medication to maintain her blood pressure. Physical examination revealed that the patient had no symptoms of dyspnea, hemoptysis, cyanosis, or abnormal sensation. Urine catecholamine and methoxyepinephrine levels were significantly increased (Table 1). An electrocardiogram (ECG) showed sinus tachycardia and echocardiography found an enlarged left atrium and a hypoechoic mass of about 4.6 cm × 4.6 cm at the top of the right atrium near the superior vena cava, with clear borders and uniform internal echogenicity. Compression of the right atrium and superior vena cava is demonstrated in Figure 1. Computed tomography (CT) examination indicated a soft tissue mass in the right atrium, about 4.7 cm × 4.4 cm in size, with a CT attenuation value of about 46 HU in plain view and a significant heterogeneous enhancement with a CT attenuation value of about 142 HU after enhancement.

**TABLE 1:** Results of laboratory test with reference values.

Variable	Value	Reference
Urine vanillylmandelic acid (μmol/24 h)	76.00	<6
Urine norepinephrine (μg/24 h)	1130.00	<70
Urine epinephrine (μg/24 h)	369.00	<20
Urea (mmol/L)	8.82	2.2–8.2
Human chorionic gonadotropin (mIU/mL)	7.68	0–5
Erythrocyte sedimentation rate (mm/h)	42.00	0–20
C-reactive protein (mg/L)	6.82	0–5
Immunoglobulin G (g/L)	16.25	5.66–14.25

**FIGURE 2:**
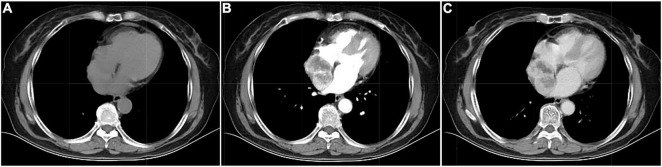
Computed tomography images of cardiac paraganglioma. **(A)** Plain image; **(B)** arterial phase image; **(C)** venous phase image.

**FIGURE 3:**
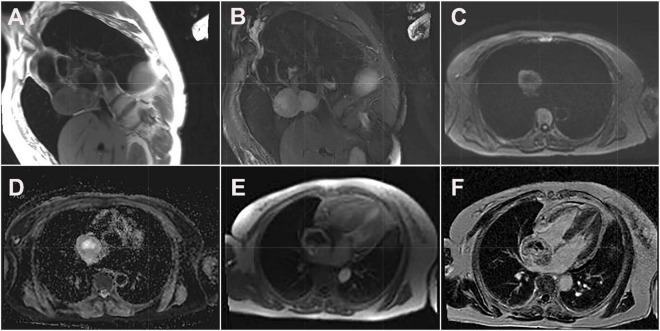
Cardiac magnetic resonance images of cardiac paraganglioma. **(A)** T1-weighted image; **(B)** T2 fat-suppressed image; **(C)** diffusion-weighted imaging; **(D)** apparent diffusion coefficient image; **(E)** first-perfused image; **(F)** delayed scans image.

**FIGURE 4:**
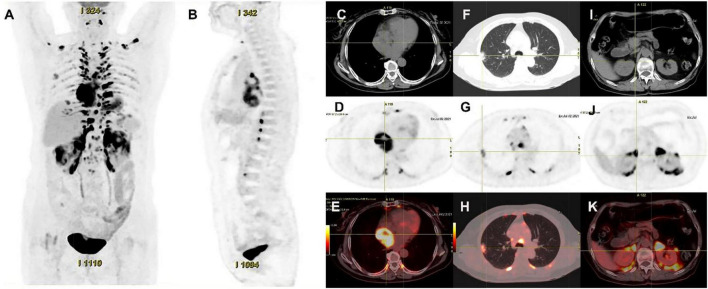
^18^F-FDG PET/CT images of cardiac paraganglioma. **(A,B)** The whole-body maximum intensity projection showed an intensely hypermetabolic thoracic mass. **(C–E)** In a cardiac lesion with a high FDG uptake, the SUVmax is about 15.2. **(F–H)** In the upper lobe of the right lung, a mixed-density nodule with a mild FDG-avid was found near the pleural, with the SUVmax at about 5.8 and a size of about 1.7 cm × 2.3 cm, inflammatory lesions were considered. **(I–K)** High FDG uptake of brown fat was demonstrated symmetrically along the perirenal and retroperitoneal area with the SUVmax at about 34.4, which was usually caused by the high secretion of catecholamine hormones from paragangliomas.

**FIGURE 5:**
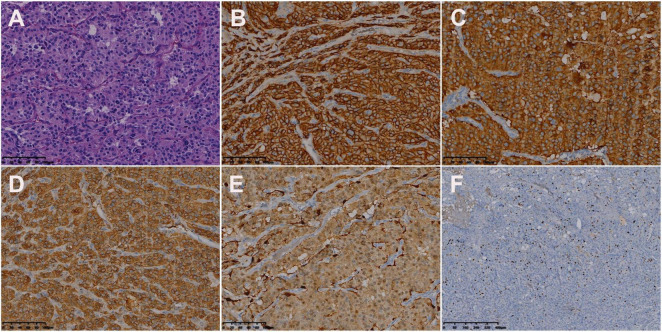
Histologic and immunohistochemical features of cardiac paraganglioma. **(A)** Hematoxylin-eosin (HE) staining showed that the tumor cells were polygonal, with round-like nuclei and rare nuclear fission images (magnification, ×200). In immunohistochemical staining, the tumor cells were positive for CD56 **(B)**, SYN **(C)**, CgA **(D)**, and S-100 **(E)**. Moreover, 3% of them were positive for Ki-67 **(F)** [magnification **(B–F)** ×200].

Its blood was supplied by the left circumflex branch of the coronary artery with a nutrient vessel visible in the lesion and a draining vein connected to the superior vena cava (Figure 2). The patient then underwent cardiac magnetic resonance (CMR) imaging. The lesion was found in the right atrium with the inferior border reaching the entrance of the inferior vena cava, with inhomogeneous iso signal on T1-weighted image (T1WI), patchy low signal inside, and inhomogeneous slightly high signal on T2 fat-suppression image, ranging from approximately 5.2 cm × 5.0 cm × 6.0 cm [Left right diameter (LR) × Anteroposterior diameter (AP) × Superior and lower diameter (SI)]. The lesion displayed inhomogeneous high signals on the diffusion-weighted imaging (DWI) and apparent diffusion coefficient (ADC) image. Heterogeneous and significant enhancement was shown in the first-perfused and delayed phases after intravenous contrast injection (Figure 3). However, abnormal signals were surprisingly found in the patient’s right lung, so an F-18 fluorodeoxyglucose-positron emission tomography/computed tomography (^18^F-FDG PET/CT) scan was performed to rule out metastatic lesions. A soft tissue mass with a high fluorodeoxyglucose (FDG) uptake was seen in the right atrium, with the maximum standardized uptake value (SUVmax) at about 15.2 and a maximum dimension of approximately 4.1 cm × 5.9 cm, which was poorly demarcated from the pericardium. In the upper lobe of the right lung, a mixed-density nodule shadow with a mild FDG uptake was seen near the pleural, with an SUVmax at about 5.8 and a size of about 1.7 cm × 2.3 cm, surrounded by a multi-density nodular shadow with a slightly radioactive uptake with an SUVmax at about 1.7. Inflammatory lesions were considered. Furthermore, high FDG uptake of brown fat was demonstrated symmetrically along the bilateral intermuscular neck, posterior cervical triangle, mediastinum, intrapericardial, sternum, spine, parasternal, perirenal, and retroperitoneal area with the SUVmax at about 34.4, which were typically related with the high secretion of catecholamine hormones from paragangliomas (Figure 4). Therefore, based on the clinical symptoms and multiple imaging findings, a CPGL in the right atrium was considered. The patient underwent a surgical resection under general anesthesia and extracorporeal circulation after preoperative blood pressure control with the α-blocker phenibutramine and metoprolol tartrate-extended release tablets. During surgery, the tumor was found to be grayish-yellowish-red in color, partially located in the atrial wall and the right atrium. The tumor had a short tip connected to the interatrial septum and a smooth and intact membrane, with a size of approximately 7.5 cm × 6.0 cm × 3.5 cm. Histopathological examination showed that the tumor cells were organoid and glandular vesicle-like in arrangement, with indistinct boundaries, an eosinophilic cytoplasm, round-like nuclei, rare nuclear fission images, abundant interstitial vessels, fibrous tissue hyperplasia, and collagenization with pigmentation. Immunohistochemistry showed Cytokeratin (CK) (−), Epithelial membrane antigen (EMA) (−), Neural cell adhesion molecules (CD56) (+), Synaptophysin (SYN) (+), Chromogranin A (CgA) (+), S-100 (+), Ki-67 (3% +), Melan-A (−), and Homatropine methyl bromide-45 (HMB45) (−). Based on the results of pathological morphology and immunohistochemistry, the tumor was diagnosed as CPGL (Figure 5). The patient showed a good prognosis after surgery with normal blood pressure and no complications or tumor recurrence.

## Discussion

We report a case of a 67-year-old female patient with primary right atrial paraganglioma and a review of the literature on imaging performed with MRI and nuclear medicine imaging. This case should be excluded from other primary cardiac tumors, such as a mucinous tumor, hemangioma, or angiosarcomas, with a different prognosis. Pheochromocytomas and paragangliomas are a series of neuroendocrine tumors originating from the adrenal medulla and chromaffin cells of the parasympathetic or sympathetic ganglia, respectively. Paragangliomas are often referred to as extra-adrenal pheochromocytomas (3). CPGL is extremely rare and occurs often in the left atrium, the left ventricle, and the left atrioventricular sulcus due to the proximity of the paraganglia cells to the left atrium and less in the right atrium, the right ventricle, and the atrial septum (4). In total, 35–50% of CPGLs secrete catecholamine hormones, which cause symptoms such as persistent or paroxysmal hypertension, headache, palpitations, and profuse sweating (5, 6). The occurrence of CPGL is associated with genetic mutations and is manifested as autosomal dominant inheritance, so the family history may provide an important diagnostic clue (7). The differentiation of benign and malignant CPGL cannot be based on the histopathological criteria but rather on the presence of distant metastases and biological behavior of recurrence (8, 9).

A literature search was performed on PubMed database from 2005 to 2022, using the keywords containing paraganglioma, cardiac, and heart. The MRI, single photon emission computed tomography (SPECT), SPECT/CT, positron emission tomography (PET)/CT, and PET/MRI manifestations of CPGL are summarized in Tables 2, 3 (1, 10–30). The purpose of imaging for CPGL is not only to localize and characterize the tumor but also to determine whether the surgical resection can be successfully undergone and to perform the post-treatment follow-up. CPGL, containing paraganglia cell nests and surrounding supporting cells, is a blood-rich tumor that often involves the coronary artery as the trophoblastic vessel. In this case, the left ileal branch is the blood-supplying artery. Echocardiography is the most common initial test. CPGL is mostly presented as a hypoechoic mass, suggesting the presence of a cardiac tumor. CPGL usually appears as round or ovoid in CT. When the tumor is large, cystic degeneration and necrosis, as well as hemorrhage, may occur. MRI is better than echocardiography and CT scan by providing more details of CPGL and its relationship to the surrounding structures. Due to the absence of radiation, MRI is more suitable for pediatric patients and screening. CPGL typically shows an equal or a low signal on T1WI but the signal will increase when the foci are accompanied by bleeding. High signals were found on T2WI with no reduction of signal in the antiphase. CPGL shows significant enhancement after contrast administration whereas extensive perfusion is seen in the early phase (31). Tumor necrosis can be detected on MRI and is a common feature seen in CPGL (23).

**TABLE 2:** Magnetic resonance imaging findings of cardiac paraganglioma.

Sr. no.	References	Age (years)	Sex	Location	T1WI	T2WI	Enhancement patter	Density characteristics
1	Duan et al. (10)	22	Woman	Left atrium	Hyperintense	Hyperintense	NA	Heterogeneous
2	Patrianakos et al. (11)	38	Man	Transverse sinus	Isointense to myocardium	Strong hyperintense	NA	NA
3	Arcos et al. (1)	22	Woman	Left atrioventricular groove	Slight hyperintense	Slight hyperintense	Focal late gadolinium enhancement	Heterogeneous
4	Berona et al. (12)	34	Woman	Left atrium	Hyperintense	Hyperintense	NA	NA
5	Degrauwe et al. (13)	43	Man	Intrapericardial	Isointense	Hyperintense	Intense enhancement	Homogenous
6	Gahremanpour et al. (14)	24	Woman	Left atrial	Hyperintense	Hyperintense	Substantial contrast enhancement	NA
7	Bhojwani et al. (15)	42	Man	Left anterior descending artery	Intermediate hyperintense	Hyperintense	Avid contrast enhancement	NA
8	Del Forno et al. (16)	46	Woman	Right atrioventricular groove	Hypointense	Hyperintense	NA	Heterogeneous
9	Beroukhim et al. (17)	16	Man	Between the aorta and the main pulmonary artery	Isointense	Hyperintense	Peripheral hyperenhancement	Heterogeneous
10	Manabe et al. (18)	46	Woman	Between the pulmonary artery trunk and the left atrium	NA	Hyperintense	Avid enhancement	Heterogeneous
11	Semionov and Sayegh, (19)	25	Woman	Right intrapericardial	NA	NA	Avid enhancement	NA
12	Tomasian et al. (20)	25	Woman	Left atrial	Hypointense	Hyperintense	Intense enhancement	Heterogeneous
13	Alghamdi et al. (21)	39	Man	Between the left atrial appendage and the main pulmonary artery	NA	NA	Avid enhancement	Heterogeneous
14	Thomas et al. (22)	32	Woman	Right atrioventricular groove	NA	NA	Avid enhancement	Heterogeneous
15	McGann et al. (23)	54	Man	Right atrium	NA	NA	Thin, circumferential rim of contrast-enhanced tissue	Heterogeneous

**TABLE 3:** The single photon emission computed tomography (SPECT) and positron emission tomography (PET) findings of cardiac paraganglioma.

Sr.no.	References	Age (years)	Sex	Location	Imaging methods	Metabolism	Distant metastasis
1	Duan et al. (10)	22	Woman	Left atrium	^18^F-FDG PET/CT	Hypermetabolic (SUVmax 29.5)	None
2	Michałowska et al. (24)	25	Man	Atrioventricular groove	^99*m*^Tc-HYNICTOC SPECT/CT	Hypermetabolic	None
3	Huot Daneault et al. (25)	47	Man	Right atrium	^18^F-FDG PET/CT	Marked hypermetabolism (SUVmax 36)	Diffuse bone metastatic
3	Huot Daneault et al. (25)	47	Man	Right atrium	^68^Ga-DOTATATE PET/CT	Hypermetabolic (SUVmax 14)	Diffuse bone metastatic
4	Wu et al. (26)	21	Man	Right atrioventricular sulcus	^18^F-FDG PET/CT	Hypermetabolic	None
4	Wu et al. (26)	21	Man	Right atrioventricular sulcus	^123^I- MIBG scintigraphy	Hypermetabolic	None
5	Arcos et al. (1)	22	Woman	Left atrioventricular groove	^18^F-FDG PET/CT	Hypermetabolic	None
6	Gahremanpour et al. (14)	24	Woman	Left atrial	^123^I-MIBG scintigraphy	Hypermetabolic	None
7	Almenieir et al. (27)	24	Woman	Right ventricle	^18^F-FDG PET/CT	Hypermetabolic	None
8	Bhojwani et al. (15)	42	Man	Abutting the left anterior descending artery	^18^F-FDG PET/MRI	Hypermetabolic (SUVmax 16)	None
9	Manabe et al. (18)	46	Woman	Between the pulmonary artery trunk and the left atrium	^123^I-MIBG scintigraphy	Obvious focal hypermetabolic	None
9	Manabe et al. (18)	46	Woman	Between the pulmonary artery trunk and the left atrium	^18^F-FDG PET/CT	Hypermetabolic (SUVmax 15.6)	None
10	Semionov and Sayegh (19)	25	Woman	Right intrapericardial	^18^F-FDG PET/CT	Hypermetabolic (SUVmax 31)	None
11	Tomasian et al. (20)	25	Woman	Left atrial	^18^F-FDG PET/CT	Hypermetabolic	None
12	Thomas et al. (22)	32	Woman	Right atrioventricular groove	^18^F-FDG PET/CT	Hypermetabolic	None
13	Dhanasopon et al. (28)	24	Woman	Left atrium	^18^F-DOPA PET/CT	Hypermetabolic	None
14	Yuan et al. (29)	15	Woman	Right atrium	^123^I-MIBG scintigraphy	Hypermetabolic	None
15	Sheehy et al. (30)	37	Woman	Right atrioventricular groove	^18^F-FDG PET/CT	Hypermetabolic	None

*MIBG, metaiodobenzylguanidine; FDOPA, 6-18F-fluoro-L-3, 4-dihydroxyphenylalanine; HYNICTOC, hydrazinonicotinyl-Tyr3-octreotide; FDG, fluorodeoxyglucose.*

For metastatic lesions, functional imaging usually has a higher sensitivity, especially for bone metastases and distant soft tissue metastases that may be difficult to visualize on CT or MRI. Radionuclide imaging techniques are available for detecting, staging, and following up on paragangliomas. Beyond their ability to detect and localize paragangliomas, these imaging methods can provide valuable additional information (32). In SPECT imaging, ^123^I-labeled metaiodobenzylguanidine (MIBG) is a guanethidine analog that is structurally similar to norepinephrine and is taken up by adrenergic storage cells in the adrenal gland and the paraganglia, allowing it to visualize neuroendocrine tissues. The sensitivity of ^131^I-MIBG scintigraphy is limited, so ^123^I-MIBG scintigraphy was introduced to improve image quality and increase sensitivity (33). Van Der Horst-Schrivers et al. (34) considered that the sensitivity of ^123^I-MIBG scintigraphy for non-metastatic and metastatic PGL has been documented to be 96 and 79%, respectively. Timmers et al. (35) researched different functional imaging methods in the localization of pheochromocytoma and paraganglioma, the sensitivity rates to the detection rate of 6-Fluoro-(18F)-l-3,4-dihydroxyphenylalanine (^18^F-DOPA PET), ^18^F-fluorodopamine PET, ^18^F-FDG PET, and ^123^I-MIBG scintigraphy in non-metastasis PGL were 81, 77, 88, and 77%, respectively, and their sensitivity rates to the detection rate of metastatic PGL were 45, 76, 74, and 57%, respectively. ^18^F-FDG PET/CT is recommended for patients with a biochemically established diagnosis of PGL when the aim is to localize the primary tumors and exclude metastases. In this case, the glucose metabolism of the soft tissue mass in the right atrium was high but metastatic lesions in the lung were excluded, which provided a powerful help for surgical resection. However, the FDG uptake alone cannot make an accurate judgment on the histological origin of the tumor so other imaging features from CMR should be integrated to further localize, characterize, and quantify the lesions.

Cardiac paraganglioma should be distinguished from a cardiac mucinous tumor, cavernous hemangioma, and angiosarcoma (10, 30). Mucinous tumors are the most common cardiac tumors mostly occurring in the left atrium with visible tips attached to the atrial septum or the atrial wall, and the lesions can follow the movement of the heart. Cardiac cavernous hemangiomas occur mostly in the left ventricle and showed hypodense on the CT scan. After enhancement, the lesions may show a gradual filling from the margin to the center. Angiosarcoma is the most common cardiac malignancy, which is mostly located in the right atrium, with dyspnea and chest pain as the main clinical manifestations. The imaging shows a mass with heterogeneous density/signal and enhancement due to the existence of necrosis. Extensive pericardial involvement and hemorrhagic pericardial effusion can be found. Due to the low incidence and variety of cardiac tumors, it is easy to miss or misdiagnose them by relying on one examination alone. Therefore, in addition to imaging methods, the diagnosis of cardiac tumors should be based on clinical and pathological examinations.

Complete surgical resection is the most effective treatment modality for CPGL; in some cases of CPGL, heart transplantation might be necessary (3). The role of adjuvant therapy in CPGL is unclear. Ayala-Ramirez et al. (36) evaluated the clinical benefit of systemic chemotherapy in patients with metastatic pheochromocytoma. The study by Tanabe et al. (37) describes the response to cyclophosphamide, vincristine, and dacarbazine (CVD) in 17 patients with malignant paraganglioma. Tumor volume or biochemical markers were decreased by only 30% in 47.1% of the patients and the progression-free survival was 31–60 months (37). Patients with complete resection of the tumor have a good long-term prognosis (38). However, more than 50% of CPGL have intraoperative or postoperative complications, whereas their proximity to the vasculature and complex surrounding tissue relationships make the procedure technically challenging, and the mortality rate is quite high (29). Adequate preoperative preparation is important to prevent intraoperative life-threatening conditions, such as a hypertensive crisis (39). This case was managed as a complete resection of the tumor under extracorporeal circulation with a good postoperative prognosis.

## Conclusion

In this case, the glucose metabolism of the right atrial soft tissue mass was high, along with pathological brown fat uptake and clinical symptoms, so CPGL was considered, which is consistent with the pathological diagnosis. The combination of ^18^F-FDG PET/CT with the CMR containing different image acquisition sequences provides a powerful aid for preoperative non-invasive diagnosis, localization, and staging of CPGL, which helps to reduce intraoperative and postoperative complications and to improve the patient prognosis. In addition, the combination of functional and anatomical imaging of PET/CT examination can help to find or exclude metastatic lesions in patients.

## Data Availability Statement

The raw data supporting the conclusions of this article will be made available by the authors, without undue reservation.

## Ethics Statement

Written informed consent was obtained from the individual(s) for the publication of any potentially identifiable images or data included in this article.

## Author Contributions

W-PH: acquisition and analysis of the work, drafting of the manuscript, data collection of imaging, and data analysis. GG and ZC: manuscript editing. Y-KQ: formal analysis and resource acquisition. J-BG: review and editing. LK: supervision and writing – review and editing. All authors contributed to the article and approved the submitted version.

## Conflict of Interest

The authors declare that the research was conducted in the absence of any commercial or financial relationships that could be construed as a potential conflict of interest.

## Publisher’s Note

All claims expressed in this article are solely those of the authors and do not necessarily represent those of their affiliated organizations, or those of the publisher, the editors and the reviewers. Any product that may be evaluated in this article, or claim that may be made by its manufacturer, is not guaranteed or endorsed by the publisher.

## References

[1] Arcos L Bustos J Acuña J Cely A Forero J Jaimes C. Cardiac paraganglioma: advantages of cardiovascular multimodality imaging. *CASE (Phila).* (2018) 2:266–72. 10.1016/j.case.2018.07.011 30582088 PMC6302035

[2] Huang W Li L Gao J Gao JB. Epithelioid hemangioendothelioma of the right atrium invaded the superior vena cava: case report and review of literature. *Int J Cardiovasc Imaging.* (2021) 37:285–90. 10.1007/s10554-020-01963-w 32812146 PMC7878244

[3] Neumann HPH Young WF Jr. Eng C. Pheochromocytoma and paraganglioma. *N Engl J Med.* (2019) 381:552–65. 10.1056/NEJMra1806651 31390501

[4] Hodin R Lubitz C Phitayakorn R Stephen A. Diagnosis and management of pheochromocytoma. *Curr Probl Surg.* (2014) 51:151–87. 10.1067/j.cpsurg.2013.12.001 24636619

[5] Tahir M Noor SJ Herle A Downing S. Right atrial paraganglioma: a rare primary cardiac neoplasm as a cause of chest pain. *Tex Heart Inst J.* (2009) 36:594–7. 20069088 PMC2801953

[6] Manger WM. An overview of pheochromocytoma: history, current concepts, vagaries, and diagnostic challenges. *Ann N Y Acad Sci.* (2006) 1073:1–20. 10.1196/annals.1353.001 17102067

[7] Young WF Jr Paragangliomas: clinical overview. *Ann N Y Acad Sci.* (2006) 1073:21–9. 10.1196/annals.1353.002 17102068

[8] Romano S Fava C Minuz P Farzaneh-Far A. Succinate dehydrogenase gene mutation with cardiac paraganglioma: multimodality imaging and pathological correlation. *Eur Heart J.* (2017) 38:1853–4. 10.1093/eurheartj/ehx007 28158325

[9] Zhang Y Wu QL Yun JP. [Interpretation of the fourth edition of WHO pathological classification of the thyroid tumors in 2017]. *Zhonghua Er Bi Yan Hou Tou Jing Wai Ke Za Zhi.* (2018) 53:718–20. 10.3760/cma.j.issn.1673-0860.2018.09.020 30293272

[10] Duan Y Xu R Liu W Cui X Li K Yang X PET/CT in a patient with cardiac paraganglioma. *Int J Cardiovasc Imaging.* (2021) 37:1473–7. 10.1007/s10554-020-02101-2 33216251

[11] Patrianakos AP Iliopoulos D Marketou M Skalidis EI Parthenakis FI. Cardiac paraganglioma: multimodality imaging of a rare tumor. *JACC Case Rep.* (2021) 3:273–5. 10.1016/j.jaccas.2020.11.047 34317516 PMC8310973

[12] Berona K Joshi R Woo YJ Shrager J. Postpartum diagnosis of cardiac paraganglioma: a case report. *J Emerg Med.* (2021) 55:e101–5. 10.1016/j.jemermed.2018.05.034 30037518

[13] Degrauwe S Monney P Qanadli SD Prior J Beigelmann-Aubry C Masci PG Intrapericardial paraganglioma: the role of integrated advanced multi-modality cardiac imaging for the assessment and management of rare primary cardiac tumors. *Cardiol J.* (2017) 24:447–9. 10.5603/CJ.2017.0091 28831780

[14] Gahremanpour A Pattakos G Reul RM Mirzai-Tehrane M. Diagnostic imaging and treatment of a left atrial paraganglioma. *Tex Heart Inst J.* (2017) 44:296–8. 10.14503/THIJ-16-5749 28878589 PMC5577961

[15] Bhojwani N Huang J Garg V Yang M Oliveira GH Rajiah P. Utility of 18F-fluorodeoxyglucose positron emission tomography/magnetic resonance imaging in the diagnosis of cardiac paraganglioma. *Indian J Nucl Med.* (2017) 32:380–2. 10.4103/ijnm.IJNM_93_17 29142369 PMC5672773

[16] Del Forno B Zingaro C Di Palma E Capestro F Rescigno G Torracca L. cardiac paraganglioma arising from the right atrioventricular groove in a paraganglioma-pheochromocytoma family syndrome with evidence of sdhb gene mutation: an unusual presentation. *Ann Thorac Surg.* (2016) 102:e215–6. 10.1016/j.athoracsur.2016.01.072 27549546

[17] Beroukhim RS del Nido P Teot LA Janeway K Geva T. Cardiac paraganglioma in an adolescent. *Circulation.* (2012) 125:e322–4. 10.1161/CIRCULATIONAHA.111.043968 22311887

[18] Manabe O Oyama-Manabe N Alisa K Hirata K Itoh K Terae S Multimodality evaluation of cardiac paraganglioma. *Clin Nucl Med.* (2012) 37:599–601. 10.1097/RLU.0b013e3182485204 22614197

[19] Semionov A Sayegh K. Multimodality imaging of a cardiac paraganglioma. *Radiol Case Rep.* (2016) 11:277–81. 10.1016/j.radcr.2016.08.002 27920843 PMC5128366

[20] Tomasian A Lai C Ruehm S Krishnam MS. Cardiovascular magnetic resonance and PET-CT of left atrial paraganglioma. *J Cardiovasc Magn Reson.* (2010) 12:1. 10.1186/1532-429X-12-1 20047692 PMC2817869

[21] Alghamdi AA Sheth T Manowski Z Djoleto OF Bhatnagar G. Utility of cardiac CT and MRI for the diagnosis and preoperative assessment of cardiac paraganglioma. *J Card Surg.* (2009) 24:700–1. 10.1111/j.1540-8191.2009.00857.x 19682163

[22] Thomas D Grant FD Kwong R Nose V Di Carli MF Dorbala S. Multimodality imaging of an unusual case of cardiac paraganglioma. *J Nucl Cardiol.* (2009) 16:644–7. 10.1007/s12350-009-9067-z 19266248

[23] McGann C Tazelaar H Cho SR Al-Saghir Y Shean F Young W In vivo detection of encapsulated intracardiac paraganglioma by delayed gadolinium enhancement magnetic resonance imaging. *J Cardiovasc Magn Reson.* (2005) 7:371–5. 10.1081/jcmr-200053642 15881516

[24] Michałowska AM ćwikła JB Konka M Kolasińska-ćwikła A Januszewicz A Michałowska I Eleven-year follow-up of cardiac paraganglioma in a patient with SDHD C11X gene mutation. *Kardiol Pol.* (2021) 79:1276–7. 10.33963/KP.a2021.0085 34392514

[25] Huot Daneault A Desaulniers M Beauregard JM Beaulieu A Arsenault F April G Highly symptomatic progressing cardiac paraganglioma with intracardiac extension treated with 177Lu-DOTATATE: a case report. *Front Endocrinol (Lausanne).* (2021) 12:705271. 10.3389/fendo.2021.705271 34367072 PMC8339957

[26] Wu C Yang X Zhang H Song Y. Cardiac paraganglioma with sulfur subunit B gene mutation: a case report. *Eur Heart J Case Rep.* (2021) 5:ytab025. 10.1093/ehjcr/ytab025 33644666 PMC7896810

[27] Almenieir N Karls S Derbekyan V Lisbona R. Nuclear imaging of a cardiac paraganglioma. *J Nucl Med Technol.* (2017) 4:247–8. 10.2967/jnmt.116.182212 28611232

[28] Dhanasopon AP Shemin RJ Yeh MW. Cardiac paraganglioma presenting as gestational hypertension. *Surgery.* (2010) 147:459–61. 10.1016/j.surg.2008.08.018 19744427

[29] Yuan WQ Wang WQ Su TW Chen HT Shi ZW Fang WQ A primary right atrium paraganglioma in a 15-year-old patient. *Endocrine.* (2007) 32:245–8. 10.1007/s12020-007-9019-9 18041591

[30] Sheehy N Kulke MH Van den Abbeele AD. F-18 FDG PET/CT in the diagnosis and management of a pericardiac paraganglioma. *Clin Nucl Med.* (2008) 33:545–6. 10.1097/RLU.0b013e31817dea57 18645373

[31] Chan EY Ali A Umana JP Nguyen DT Hamilton DJ Graviss EA Management of primary cardiac paraganglioma. *J Thorac Cardiovasc Surg.* (2020) S0022-5223:32704–5. 10.1016/j.jtcvs.2020.09.100 33148444

[32] Taïeb D Jha A Treglia G Pacak K. Molecular imaging and radionuclide therapy of pheochromocytoma and paraganglioma in the era of genomic characterization of disease subgroups. *Endocr Relat Cancer.* (2019) 26:R627–52. 10.1530/ERC-19-0165 31561209 PMC7002202

[33] Jalil ND Pattou FN Combemale F Chapuis Y Henry JF Peix JL Effectiveness and limits of preoperative imaging studies for the localisation of pheochromocytomas and paragangliomas: a review of 282 cases. French association of surgery (AFC), and the French association of endocrine surgeons (AFCE). *Eur J Surg.* (1998) 164:23–8. 10.1080/110241598750004913 9537705

[34] Van Der Horst-Schrivers AN Jager PL Boezen HM Schouten JP Kema IP Links TP. Iodine-123 metaiodobenzylguanidine scintigraphy in localising phaeochromocytomas–experience and meta-analysis. *Anticancer Res.* (2006) 26:1599–604. 16619578

[35] Timmers HJ Chen CC Carrasquillo JA Whatley M Ling A Havekes B Comparison of 18F-fluoro-L-DOPA, 18F-fluoro-deoxyglucose, and 18F-fluorodopamine PET and 123I-MIBG scintigraphy in the localization of pheochromocytoma and paraganglioma. *J Clin Endocrinol Metab.* (2009) 94:4757–67. 10.1210/jc.2009-1248 19864450 PMC2795662

[36] Ayala-Ramirez M Feng L Habra MA Rich T Dickson PV Perrier N Clinical benefits of systemic chemotherapy for patients with metastatic pheochromocytomas or sympathetic extra-adrenal paragangliomas: insights from the largest single-institutional experience. *Cancer.* (2012) 118:2804–12. 10.1002/cncr.26577 22006217 PMC3882190

[37] Tanabe A Naruse M Nomura K Tsuiki M Tsumagari A Ichihara A. Combination chemotherapy with cyclophosphamide, vincristine, and dacarbazine in patients with malignant pheochromocytoma and paraganglioma. *Horm Cancer.* (2013) 4:103–10. 10.1007/s12672-013-0133-2 23361939 PMC10358011

[38] Khan MF Datta S Chisti MM Movahed MR. Cardiac paraganglioma: clinical presentation, diagnostic approach and factors affecting short and long-term outcomes. *Int J Cardiol.* (2013) 166:315–20. 10.1016/j.ijcard.2012.04.158 22652039

[39] Chen G Wang J Weinberg L Robinson C Ho T Lin W Anaesthetic management of cardiac phaeochromocytoma: a case series. *Int J Surg Case Rep.* (2018) 51:134–8. 10.1016/j.ijscr.2018.08.019 30153610 PMC6110996

